# Early neurovascular changes in the retina in preclinical diabetic retinopathy and its relation with blood glucose

**DOI:** 10.1186/s12886-021-01975-7

**Published:** 2021-05-17

**Authors:** Hui Li, Xiaobing Yu, Bodi Zheng, Shan Ding, Zhongqing Mu, Lixin Guo

**Affiliations:** 1 Department of Endocrinology, Beijing Hospital, Nations Center of Gerontology, Beijing, China; 2 Institute of Geriatric Medicine, Chinese Academy of Medical Sciences, P.R.China, Beijing, China; 3 Department of Ophthalmology, Beijing Hospital, Nations Center of Gerontology, Beijing, China

**Keywords:** Vessel density, Acircularity index, Retinal nerve fiber layer thickness, Retinopathy, Type 2 diabetes

## Abstract

**Background:**

To investigate the changes in retinal nerve fiber layer thickness and macular blood flow density during the preclinical stage of diabetic retinopathy and their relationship with blood glucose.

**Methods:**

In this cross-sectional study, 97 diabetic patients (total of 188 eyes; 144 eyes in no diabetic retinopathy group, 44 eyes in mild diabetic non-proliferative retinopathy group) and 35 healthy people (70 eyes) were enrolled, All the subjects were divided into different groups based on their HbA1c levels, and they underwent optical coherence tomography angiography. We compared the optical coherence tomography angiography parameters and retinal nerve fiber layer thickness among the different glucose groups.

**Results:**

The parafoveal vessel density and the temporal retinal nerve fiber layer thickness were lower (*p* < 0.05) in the diabetic group than in the normal group. The diabetic group showed a higher acircularity index than the normal group. The normal group had the highest vessel density and the lowest acircularity index, followed by the no-diabetic retinopathy group and the mild non-proliferative retinopathy group, (*p* < 0.001). Foveal vascular density and parafoveal vessel density decreased with an increase in HbA1c. There was a negative correlation between parafoveal vessel density in the deep retinal vascular layer and fasting blood glucose (*p* < 0.01). The temporal retinal nerve fiber layer thickness decreased across the HbA1c level groups, and was positively correlated with the parafoveal vessel density in the superficial retinal vascular layer (*p* < 0.05).

**Conclusions:**

This study shows that retinal microvasculopathy and neuropathy can be present in the absence of retinopathy. The vessel density of the deep retinal vascular layer was negatively correlated with fasting blood glucose, and the temporal retinal nerve fiber layer thickness was positively correlated with the vessel density of the superficial retinal vascular layer. These indicators are helpful for endocrinologists and ophthalmologists in detecting early diabetic retinal pathological lesions.

## Background

Diabetic retinopathy (DR) is a diabetes-specific microangiopathy, which is one of the main causes of blindness globally. It affects more than one-third of patients with diabetes and has gradually become a global public health challenge [1–3]. Recent studies have found that DR manifests in both microvasculopathy and neuropathy. The neurodegenerative changes in DR include apoptosis of retinal neurons and thinning of the retinal nerve fiber layer (RNFL) thickness [4, 5]. The RNFL is composed of retinal ganglion cell axons, and it makes up the innermost neural layer of the retina. Changes in RNFL thickness reflect optic nerve injury. The microangiopathy of DR may manifest as a decrease in capillary density within the macular area. The macular fovea is the most acutely visual area, and it is surrounded by a capillary arch. The central avascular area is called the foveal avascular zone (FAZ), which is important for maintaining fine vision. The changes in the morphological area of the FAZ and the vessel density of the surrounding capillary network may reflect the degree of retinal ischemic disease. Optical coherence tomography angiography (OCTA) can simultaneously show the morphology and density of macular vessels and measure RNFL thickness. The purpose of this study was to investigate the changes in RNFL thickness of the retinal optic disc and the macular blood flow density during the preclinical stage of diabetic retinopathy and their relationship with blood glucose levels.

## Methods

### Participants

This cross-sectional study was performed at the endocrinology department of Beijing Hospital and approved by the Institutional Ethics Committee of Beijing Hospital. The participants were recruited from February 2018 to December 2019. The subjects signed an informed consent form, and any accompanying images were obtained from all participants. The diagnosis of type 2 diabetes mellitus was based on the 1999 WHO standards. The inclusion criteria were as follows: (1) best-corrected Snellen visual acuity of 20/200 or better; (2) intraocular pressure (IOP) within10–21 mmHg; (3) no history of external injury or internal and external eye surgery; (4) no other eye lesions except mild cataract. The exclusion criteria were as follows: (1) age-related macular degeneration; (2) congenital maculopathy; (3) macular epiretinal membrane; (4) keratopathy, lens opacity, vitreous liquefaction, or hemorrhage; (5) other non-diabetic chorioretinopathy (e.g.,hypertensive retinopathy); (6) refractive error > 3.00 D; (7) severe non-proliferative diabetic retinopathy (NPDR)and proliferative diabetic retinopathy (PDR); (8) retinal choroiditis and uveitis; (9) glaucoma, including acute angle-closure glaucoma, chronic angle-closure glaucoma and primary open-angle glaucoma, and history of elevated IOP; (10) retinal arteriovenous occlusion; (11) photocoagulation or eye surgery. Initially, 117 patients with type 2 diabetes were enrolled, and 20 patients with incomplete data were excluded. Finally, data from 97 patients were included for the statistical analysis. Among them, 62 were male and 35 were female, with an average age of 54 ± 10 years, fasting blood glucose (FBG) of 8.0 ± 2.6 mmol/L, and HbA1c of 9.1 ± 1.9%. The normal control group consisted of 35 healthy staff and volunteers without diabetes or ophthalmological diseases. There were 10 males and 25 females, with an average age of 53 ± 11 years, FBG of 5.2 ± 0.4 mmol /L, and HbA1c of 5.6 ± 0.3%. According to the HbA1c level quartile, patients with diabetes were divided into four groups: HbA1c = 6.2–7.7%, *N* = 52; HbA1c = 7.7–8.9%, *N* = 44; HbA1c = 8.9–10.1%, *N* = 46; HbA1c > 10.1%, *N* = 46. The normal controls were considered a separate group (HbA1c < 6.1%).

### Ocular examination

Routine dilated fundus examinations, including simple funduscopy and fundus photography were performed by fixed ophthalmologists, and the fundus lesions, excluding PDR, moderate and severe NPDR and macular edema, were assessed according to the Early Treatment Diabetic Retinopathy Study (ETDRS) international DR staging standard [6]. All patients underwent IOP measurement, and they were divided into no-DR and mild NPDR groups.

### Optical coherence tomography angiography imaging

All OCTA examinations were performed by the same operator. The examiners took the sitting position and adjusted their eyes to the appropriate position without dilating the pupil. OCTA of the macular area was performed using an AngioVue OCTA device (Optovue, Inc. RTVue XR), which has an A-scan rate of 70,000 scans per second, a wavelength of 840 nm, and a bandwidth of 45 nm, with each OCTA volume containing 304 × 304 A scans and two consecutive B scans captured at each fixed position. The scanning area was a 3 × 3-mm section centered on the fovea, and two orthogonal OCTA scans were acquired to minimize motion artifacts.

The newly developed built-in AngioAnalytics software (version 2017.1.0.155; Optovue, Inc.) was used to quantitatively evaluate vessel density (VD) of superficial and deep capillaries of the retina, FAZ area size, and RNFL thickness. An image quality index (QI) ranging from 0 to 10 was provided by the software for each scan. The capillary plexus within the macular region was automatically stratified into two layers: the superficial retinal vascular layer extending from the inner limiting membrane to the inner plexiform layer with an offset of 10 mm and the deep retinal vascular layer from the inner plexiform layer with an offset of 10 mm to the outer plexiform layer with an offset of 10 mm. The whole VD indicates the vessel density of the entire image. The parafoveal region is defined as a 3.0-mm-wide round annulus around the fovea 1.0 mm circle, and the foveal vascular density (FD-300) was determined as the vessel density within a ring with a width of 300 μm around the FAZ. The FAZ measurements were generated based on the retina slab with automated detection of the FAZ boundary using the AngioVue software. The acircularity index (AI) was calculated as the ratio of the FAZ perimeter divided by the perimeter of a circle with an equal area. Using the optic disc as the center, RNFL thickness was measured using a circular scan with a diameter of 3.46 mm, including the superior, inferior, nasal and temporal sides of the optic disc. All analyses were performed by the same investigator.

There were 188 eyes in the diabetic group, including 144 eyes in the no-DR group and 44 eyes in the mild NPDR group; the other six eyes were not further grouped because their QIs were less than 6. Seventy eyes in the control group.

### Statistical analysis

The statistical analyses were performed using SPSS software for Windows (version 16.0). All data are shown as the mean ± standard deviation (xˉ ± s). Differences in the data were assessed using the independent t-test or analysis of variance (ANOVA), and pairwise comparisons were made using one-way ANOVA with post-hoc pairwise comparison. The mixed effect model was used to verify the results. Spearman rank correlation analysis was used to analyze the correlation between VD, blood glucose and RNFL. Statistically significance was set at *p* < 0.05.

## Results

### Patients characteristics

This study included 188 eyes from 97 patients with type 2 diabetes. Six eyes were excluded because of QIs less than 6. There were 144 eyes (72 patients) in the no-DR group and 44 eyes (25patients) in the mild NPDR group. Seventy eyes of 35 age-matched healthy controls were also included. The mean ages of the control group, no-DR group and mild NPDR group were 53 ± 11, 53 ± 10, and 57 ± 9 years, respectively. The mean duration of diabetes was 9.2 ± 6.0 years for the no-DR group and 14.4 ± 9.1 years for the mild NPDR group (*p* < 0.05). There was a significant difference between the HbA1c levels of the two groups (9.1 ± 1.9% versus 5.6 ± 0.3%, in the diabetic and control groups, *p* < 0.001). Systolic blood pressure (SBP) in the diabetic group was higher than that in the control group (128 ± 19 mmHg vs. 116 ± 8 mmHg, *p* < 0.001). There was no difference between the creatinine and total cholesterol levels of the diabetic and control groups. (Table 1).

**Table 1:** Characteristics of the Study Participants

	DM					Control group		
	no-DR (*N* = 72)	mild NPDR (*N* = 25)	*t*	p*	Total (*N* = 97)	*N* = 35	*T*	P**
Age (years)	53 ± 10	57 ± 9	1.725	0.088	54 ± 10	53 ± 11	0.586	0.559
DM duration (years)	9.2 ± 6.0	14.4 ± 9.1	2.694	***0.011***	10.5 ± 7.3	*NA*	*NA*	*NA*
SBP (mmHg)	127 ± 12	136 ± 19	2.386	***0.023***	128 ± 19	116 ± 8	5.017	***< 0.001***
DBP (mmHg)	79 ± 10	80 ± 9	0.482	0.630	79 ± 10	72 ± 14	8.545	***< 0.001***
IOP (mmHg)	16.9 ± 2.1	17.1 ± 1.9	0.149	0.882	17.0 ± 2.0	16.6 ± 1.9	1.490	0.138
HbA1c (%)	8.9 ± 1.8	9.5 ± 2.2	1.186	0.239	9.1 ± 1.9	5.6 ± 0.3	9.102	***< 0.001***
Fasting blood Glucose (moll/l)	8.1 ± 2.7	7.9 ± 2.6	−0.391	0.696	8.0 ± 2.6	5.2 ± 0.4	9.728	***< 0.001***
Cr (umol/l)	60.2 ± 24.8	70.7 ± 32.0	1.658	0.101	63 ± 27	55 ± 11	1.463	0.146
TC (mmol/l)	4.1 ± 1.0	4.1 ± 1.1	0.001	1.0	4.1 ± 1.1	4.4 ± 0.9	−1.584	0.116

*SBP* Systolic blood pressure, *DBP* Diastolic blood pressure, *IOP* Intraocular pressure, *Cr* Creatinine, *TC* Total cholesterol

* Comparison of the no-DR and mild DR groups using the independent samples t-test

** Comparison of the DM (total) and control groups using the independent samples t-test

### Comparison of the OCTA parameters and RNFL thickness in the diabetic and the normal control groups

**Fig. 1:**
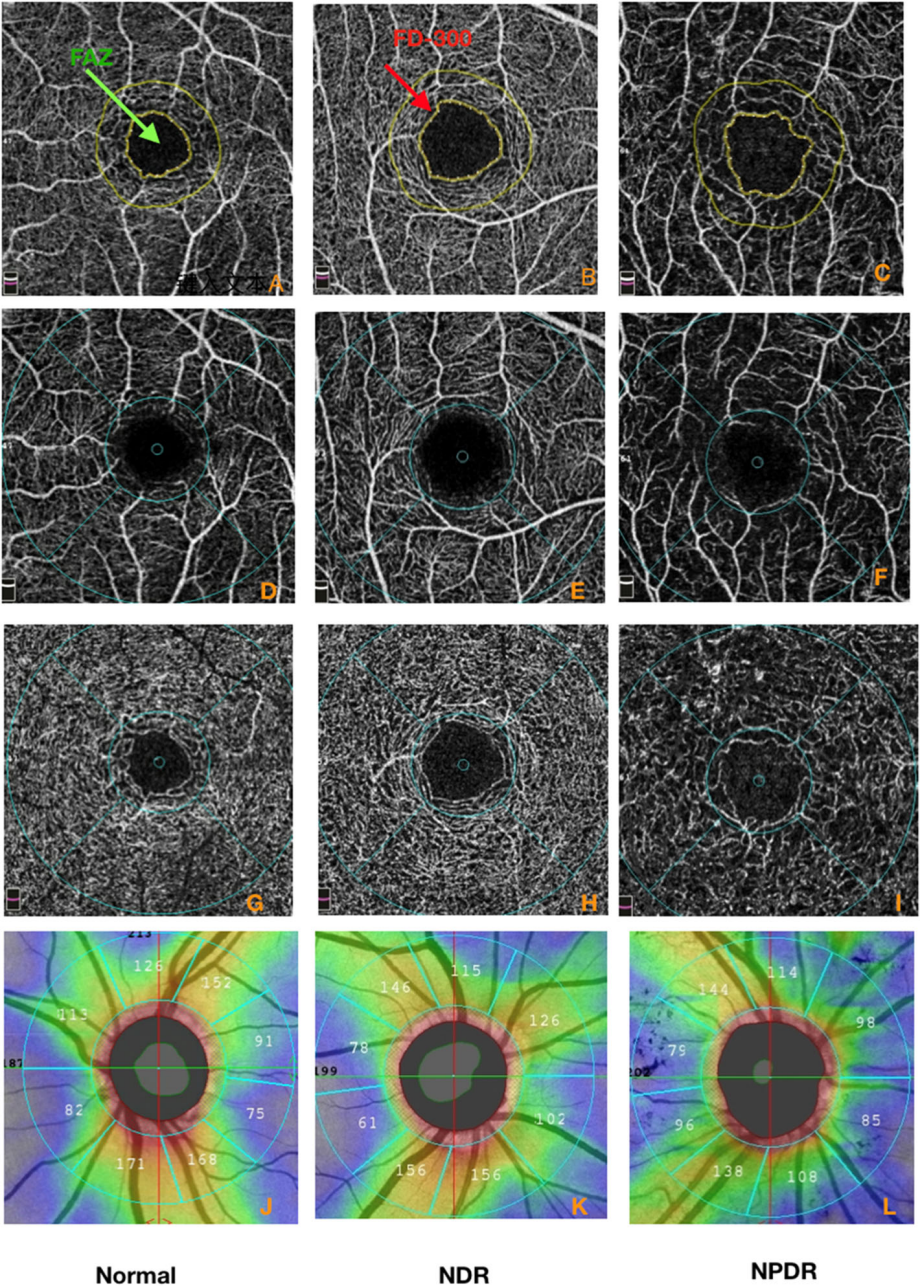
Representative sample of vessel densities in the macula region and RNFL thickness of optic disc for each group (control, NDR, mild NPDR) .green arrow indict FAZ, red arrow indict FD-300, Top row: the size and shape of FAZ, FD-300; Second row: VD of the superficial retinal vascular layer in macular area; Third row: VD of the deep retinal vascular layer in macular area; Bottom row: RNFL thickness in optic disc

The diabetic group showed a significantly (*p* < 0.001) lower VD in the superficial and deep retinal vascular layers in the parafoveal and whole region than the normal group. Additionally, the FD-300 was significantly (*p* < 0.001) lower in the diabetic group than in the normal group. The AI value in the diabetic group was significantly higher than that in the normal control group. The area of the FAZ in the diabetic group was slightly larger than that in the normal control group, but there was no significant difference between the two groups (*p* = 0.583). From the normal control group to the no-DR group and then to the mild NPDR group, the VD of the superficial and deep retinal vascular layer and FD-300 decreased gradually, and the difference was significant. The one-way ANOVA with post hoc pairwise comparison showed that there were significant differences between the groups (*p* < 0.001). The size of the FAZ did not significantly differ (*p* = 0.659) in the three groups. AI increased gradually, and the three groups showed significant differences (1.14 ± 0.05 vs. 1.16 ± 0.06vs1.20 ± 0.08, F = 13.101, *p* < 0.001).(Table 2 and Fig. 1).

**Table 2:** Comparisons of OCTA parameters in retina in the normal control and diabetes with or without retinopathy groups

Number	DM				Control group		
	no-DR (*N* = 144)	Mild NPDR (N = 44)	P*	Total (*N* = 188)	*N* = 70	P**	p***
Superficial Whole VD(%)	44.4 ± 4.1	40.4 ± 5.2	***< 0.001***	43.4 ± 4.7	46.5 ± 2.6	***< 0.001***	***< 0.001***
Superficial parafoveal VD(%)	47.3 ± 4.4	42.8 ± 5.5	***< 0.001***	46.3 ± 5.1	49.4 ± 3.1	***< 0.001***	***< 0.001***
Deep whole VD(%)	49.1 ± 3.7	45.3 ± 4.5	***< 0.001***	48.2 ± 4.2	51.2 ± 7.1	***< 0.001***	***< 0.001***
Deep parafoveal VD(%)	51.7 ± 3.9	47.6 ± 5.0	***< 0.001***	50.7 ± 4.5	53.8 ± 7.4	***< 0.001***	***< 0.001***
FD-300(%)	47.7 ± 4.6	43.5 ± 5.4	***< 0.001***	46.8 ± 5.1	51.2 ± 3.5	***< 0.001***	***< 0.001***
FAZ Area (μm^2^)	0.35 ± 0.17	0.38 ± 0.13	0.466	0.36 ± 0.16	0.37 ± 0.28	0.583	0.659
AI	1.16 ± 0.06	1.20 ± 0.08	***< 0.001***	1.17 ± 0.06	1.14 ± 0.05	***< 0.001***	***< 0.001***

*VD* Vessel density, *FD-300* The foveal density, *AI* The acircularity index

*Comparison of the no-DR and mild NPDR groups using One-way ANOVA with post hoc pairwise comparison

**Comparison of the DM (total) and control groups using the independent samples t-test

***Comparison of the no-DR, mild NPDR and control groups using one-way ANOVA

The RNFL thickness in the superior and temporal sides of the optic disc was significantly lower in the diabetic group than in the normal group (*p* < 0.05). Interestingly, the temporal RNFL thickness of the optic disc of the normal and mild NPDR groups were significantly thicker than that of the no-DR group, but the superior, inferior and nasal RNFL thickness were not significantly different. (Table 3 and Fig. 1).

**Table 3:** Comparisons of RNFL thickness in disc in the normal control and diabetes with or without retinopathy groups

	DM				Control group		
	no-DR (N = 144)	Mild NPDR (*N* = 44)	p*	Total (*N* = 188)	N = 70	p**	p***
Temporal (μm)	72.5 ± 10.4	78.6 ± 12.9	***0.001***	73.9 ± 11.3	78.1 ± 10.6	***0.008***	***< 0.001***
Nasal (μm)	76.2 ± 11.8	76.8 ± 12.3	0.802	76.4 ± 11.9	74.7 ± 16.9	0.459	0.665
Superior (μm)	122.8 ± 16.7	122.5 ± 22.1	0.918	122.7 ± 18.1	128.4 ± 15.3	***0.020***	0.068
Inferior (μm)	129.5 ± 15.8	129.9 ± 19.9	0.897	129.6 ± 16.8	132.9 ± 19.1	0.171	0.389

Temporal, the RNFL thickness in temporal field of disc; Nasal, the RNFL thickness in nasal field of disc; Superior, the RNFL thickness in superior field of disc; Inferior, the RNFL thickness in inferior field of disc.

*Comparison of the no-DR and mild NPDR groups using One-way ANOVA with post hoc pairwise comparison

**Comparison of the DM (total) and control groups using the independent samples t-test

***Comparison of the no-DR, mild NPDR and control groups using one-way ANOVA

Because of the binocular datas, we considered the correlation between the two eyes, and the mixed effect model,based on the participants as the main body and the eyes as duplicates, was used for verification. The results showed that there were still significant differences in blood flow density, FD-300, AI, and temporal RNFL thickness in the normal control group and the diabetic group, as well as between the normal control, no-DR and mild NPDR groups (*p* < 0.05, 0.01, respectively).

Because of the significant difference of SBP in groups, we used SBP as a covariate, grouping as a fixed factor, and VD and RNFL as dependent variables. The results showed that SBP had no significant effect on the VD or RNFL. (Table 4) Covariance analysis, using DBP as a covariate, also showed that DBP had no significant effect on blood flow density and RNFL.

**Table 4:** Covariance results of VD and RNFL

Dependent variable	Fixed factor	Covariate	p*	p**
Superficial Whole VD(%)	Group (DM vs Control)	SBP	***< 0.001***	0.533
Superficial parafoveal VD(%)			***< 0.001***	0.491
Deep whole VD(%)			***< 0.001***	0.525
Deep parafoveal VD(%)			***< 0.001***	0.618
FD-300(%)			***< 0.001***	0.861
AI			***< 0.01***	0.673
Temporal (μm)			***< 0.05***	0.997
Superior (μm)			***< 0.05***	0.634

*The independent effect of fixed factor on dependent variables

**The effect of covariate on dependent variables

### Analysis of OCTA parameters and RNFL thicknesses in different blood glucose level groups

After grouping the participants into four according to the quartile of the HbA1c levels, we observed that with the increase in HbA1c, FD-300 and VD gradually decreased from the control group to the third group, but VD and FD-300 in the fourth group were slightly higher than those in the third group. Multiple comparisons revealed statistically significant differences in the OCTA parameters of the control group and the other four groups (*p* < 0.01), but there were no statistically significant differences across the other four groups (*p* > 0.05). The AI of the control group was significantly lower than the other four groups (*p* < 0.01). There was no significant difference in the FAZ size in the groups (*p* > 0.05). The temporal RNFL thickness of the optic disc decreased gradually among groups with different HbA1c levels, but abnormally increased in group 3 (Table 5).

**Table 5:** Comparisons of OCTA parameters of retina and the RNFL thickness of disc in 5 groups of the different HbA1c levels

	Control group (< 6.1%)	Group1 6.2% ~ 7.7%)	Group2 (7.7% ~ 8.9%)	Group3 (8.9% ~ 10.1%)	Group4 (> 10.1%)	p***
Number	70	52	44	46	46	
Superficial Whole VD(%)	46.5 ± 2.6*	43.9 ± 4.6	43.2 ± 4.8	43.2 ± 4.9	43.3 ± 4.4	***< 0.001***
Superficial parafoveal VD(%)	49.4 ± 3.1*	47.0 ± 4.9	45.8 ± 5.5	45.8 ± 5.2	46.1 ± 4.6	***< 0.001***
Deep whole VD(%)	51.2 ± 7.1*	48.4 ± 3.2	48.1 ± 3.9	47.5 ± 4.8	48.8 ± 4.9	***< 0.01***
Deep parafoveal VD(%)	53.8 ± 7.4*	50.9 ± 3.2	50.7 ± 4.1	49.9 ± 5.3	51.4 ± 5.3	***< 0.01***
FD-300(%)	51.2 ± 3.5*	47.2 ± 5.3	46.0 ± 4.7	46.6 ± 4.9	47.3 ± 5.2	***< 0.001***
FAZ Area (um2)	0.37 ± 0.28	0.38 ± 0.22	0.33 ± 0.10	0.33 ± 0.13	0.37 ± 0.14	0.556
AI	1.14 ± 0.05*	1.16 ± 0.05	1.17 ± 0.09	1.17 ± 0.07	1.16 ± 0.05	***< 0.01***
Temporal (um)	78.1 ± 10.6**	74.4 ± 9.3	71.3 ± 9.5	77.6 ± 11.2**	71.2 ± 11.4	***< 0.01***
Nasal (um)	74.7 ± 16.9	79.3 ± 9.8	74.5 ± 13.0	74.9 ± 13.5	76.3 ± 10.7	0.328
Superior (um)	128.4 ± 15.3	125.5 ± 17.1	119.5 ± 19.7	123.7 ± 16.7	120.4 ± 17.3	0.165
Inferior (um)	132.9 ± 19.1	130.9 ± 13.4	127.7 ± 18.7	128.3 ± 18.3	131,3 ± 14.7	0.497

*Comparison of the Control and other groups by One-way ANOVA with post hoc pairwise comparison with p < 0.01

*Comparison of the group2,4 and other groups by One-way ANOVA with post hoc pairwise comparison with p < 0.05

***Comparison of 5 groups by one-way ANOVA

Spearman correlation analysis showed that there was a significant negative correlation between parafoveal, whole vessel density of the deep retinal layer, and FBG (r = − 0.170, *p* = 0.006; r = − 0.163, *p* = 0.009, respectively). RNFL thickness in temporal optic disc was positively correlated with parafoveal VD in superficial layer (r = 0.131, *p* < 0.05).

## Discussion

In this study, we analyzed the changes in macular blood flow density, FAZ area, and RNFL thickness of the optic disc in the normal control, no-DR and mild NPDR groups using OCTA, and we determined whether they were correlated with blood glucose levels. Diabetic retinopathy (DR) is a specific diabetic microvascular complication. Hyperglycemia leads to retinal microvascular disorder, non-perfusion in the capillary plexus and macular ischemia. In the past, we found microaneurysms during fundus fluorescein angiography to diagnose early diabetic retinopathy, but several recent studies have reported changes in the retinal capillaries before the clinical diagnosis of diabetic retinopathy, including a decrease in parafoveal VD and irregular FAZ morphology [7–9]. Our results are consistent with those findings. Compared with the control group, the VD of the superficial and deep retinal layers and FD-300 were significantly lower in the no-DR group. These results suggest that even if diabetic retinopathy has not been diagnosed clinically, the blood density of the retinal capillary network, especially the density around the fovea, was impaired, and this represented the low perfusion during the preclinical diabetic retinopathy stage. Previous studies have also suggested that parafoveal VD has a good correlation with diabetic microvascular disease [10], which may be used as a marker of pre-diabetic retinopathy [9].

Regarding the changes in the FAZ area in diabetic patients, the results were not consistent. Some studies have shown that there is no significant change in the FAZ area in diabetic patients without retinopathy [7–9], while other studies have found that the FAZ area in diabetic patients without retinopathy is significantly higher than that in normal people [11–14]. Previous studies have shown that there are individual differences in the FAZ area [15, 16], and the AUC area of the FAZ is small, so it is difficult to determine whether FAZ is pathologically enlarged [17]. Therefore, FAZ may not be a sensitive marker for the diagnosis of early diabetic retinopathy. The irregular shape of the FAZ caused by the decrease of capillary network perfusion and macular ischemia may better reflect ischemia in the fovea. Fang used the FAZ circularity index to evaluate the FAZ shape, and the result showed that the FAZ circularity index decreased in DR. [18] AI is a new index for evaluating the degree of capillary injury in the FAZ area, which reflects the morphological change in the FAZ and the degree of capillary circulation around the fovea following tortuous changes with the FAZ perimeter. Compared with other FAZ indexes, AI can better reflect the irregularity of FAZ morphology. The results of our study are the first to show that the AI increases gradually from the normal control group across the no-DR group and the mild NPDR group. This indicated that the FAZ morphology of the no-DR group changed irregularly, and the FAZ morphology of the mild NPDR group was more irregular, indicating the aggravation of ischemia.

Blood glucose levels affect retinal blood VD. The study has shown that low VD around the optic disc is related to high FBG levels [8]. Lavia found a rapid decrease in macular VD over 12 months that was associated with a rapid improvement in blood glucose levels [19]. Among the groups with different HbA1c levels, we found that there were significant differences between the VDs of the control group and other groups, but there was no significant difference among the other four groups. It was speculated that the retinal microcirculation showed significant blood flow has changed at the beginning of the increase in blood glucose level, and the VD further decreased with an increase in blood glucose level, but the change was not significant. The results suggest that the initial stages of the elevation of the blood glucose level are the most effective for intervention. However, our results require a large sample size to confirm. OCTA parameter alterations for the deep vascular layer are more serious than those for the superficial vascular layer in NPDR [20–23], especially in patients with DME [24–26]. Our results showed that the VD of the deep retinal layers in the macular area was negatively correlated with the FBG level; the VD in the superficial layer of the retina was not correlated, suggesting that the deep retinal VD was more closely related to the change in blood glucose.

The neurons, ganglion cell layer, and nerve fiber layer of the inner retina are closely connected with the extensive capillary network and form functional neurovascular units together with glial cells, pericytes, and capillary endothelial cells. The nerve fiber layer is composed of axons of optic ganglion cells, which are mostly unmyelinated axons, that require more energy and are more vulnerable to ischemic damage [27]. The blood supply to these nerve tissues mainly originates from the superficial capillary network of the retina. During the preclinical DR and the early stages of diabetic retinopathy, microvascular changes are accompanied by retinal neurodegenerative and neuroelectrophysiological changes [4, 5, 9]. Thinning of RNFL and GC-IPL suggests neurodegenerative changes, and it is related to diabetic retinopathy [28, 29]. Although one study showed that there were no significant changes in the average or sectoral RNFL thicknesses in patients without DR [30], other studies found that RNFL loss may occur in patients without DR. [31, 32] Our study showed that the temporal RNFL in diabetic patients was significantly thinner than that in the normal group. Further analysis revealed that the temporal RNFL of the no-DR group was significantly thinner than that of the normal group. The decrease in RNFL thickness indicates that the retinal nerve tissue undergoes degenerative changes during the preclinical DR stage. We also found that the temporal RNFL thickness of the optic disc was only positively correlated with parafoveal VD in the superficial retinal vascular layer. The RNFL is located within the superficial capillary network of the retina, and the macular fovea is located on the temporal side of the optic disc, therefore, changes in the parafoveal VD in the superficial retina of the macular area may affect the temporal RNFL thickness of the optic disc. This suggests that microangiopathy and neuropathy work together in retinopathy, and they are related to each other.

Poor blood glucose control can lead to the decrease in RNFL thickness. A previous study showed that poorer glucose tolerance was significantly associated with the reduction in RNFL, but it did not show a significant correlation with HbA1c [33]. In our study, there was a significant difference in the temporal RNFL thickness among the different HbA1c level groups, which decreased gradually, but abnormally increased in group 3. There was no significant correlation between RNFL thickness and HbA1c levels. Local leakage worsened with the development of hyperglycemia. If severe microvascular lesions such as macular edema, occurs, they are more likely to cause damage to the deep capillary network of the retina [24], and the intracellular and extracellular edema can cause an abnormal increase in RNFL thickness. As a result, there was no significant correlation between HbA1c level and RNFL thickness.

This study has some limitations in this study. First, this study is a small cross-sectional clinical study, which is not comprehensive enough, and the findings need to be further confirmed by a study with a larger sample. Second, although changes in retinal VD and RNFL thickness were observed simultaneously, and the relationship between VD and blood glucose levels was also observed, it is still not clear whether there is a correlation between RNFL thickness and blood glucose changes. This may be attributed to our small samples.

In summary, our studies have shown that there is a decrease in macular vessel density and thinning of the temporal RNFL of the optic disc during the preclinical DR stage. The macular superficial and deep VDs decreased and AI increased gradually from the normal group across the no-DR group and the mild NPDR group, respectively. The deep macular VD was negatively correlated with FBG, while the temporal RNFL thickness of the optic disc was positively correlated with the superficial macular VD. Attention should be paid to the early changes in retinal microvascular and neurodegeneration in T2DM without DR. Indicators, such as macular VD, AI, and the temporal RNFL thickness of the optic disc, can help us to detect early pathological changes. The changes in VD associated with different blood glucose levels underscore the importance of early intervention and treatment.

## Data Availability

The datasets used and/or analyzed during the current study are available from the corresponding author on reasonable request.
